# The Best Timing of Mate Search in *Armadillidium vulgare* (Isopoda, Oniscidea)

**DOI:** 10.1371/journal.pone.0057737

**Published:** 2013-03-01

**Authors:** Fanny Beauché, Freddie-Jeanne Richard

**Affiliations:** Université de Poitiers, Laboratoire Ecologie et Biologie des Interactions, UMR CNRS 7267, Equipe Ecologie Evolution Symbiose, Poitiers, France; University of York, United Kingdom

## Abstract

Mate choice is mediated by many components with the criteria varying across the animal kingdom. Chemical cues used for mate attractiveness can also reflect mate quality. Regarding the gregarious species *Armadillidium vulgare* (isopod crustacean), we tested whether individuals can discriminate conspecifics at two different levels (between sex and physiological status) based on olfactory perception. Tested conspecifics were individuals of the same or opposite sex, with the females at different moult stages. We found that the attractiveness of individuals was mediated by short-distance chemical cues and tested individuals were able to discriminate and prefer individuals of the opposite sex. Moreover, male preference to female increased during their moulting status as they matured. Males were particularly more attracted by females with appearing white calcium plates, which corresponds to the beginning of their higher receptivity period. These differences in attractiveness due to sex and physiological status are likely to shape the composition of aggregates and facilitate mate finding and optimize the reproductive success for both males and females. Thus aggregation pheromones could be linked to sex pheromones in terrestrial isopods.

## Introduction

### Mate Finding and Cues

As mates are not always receptive, mate finding should not include only locating a mate but also doing so with good timing. Indeed, the ability to discriminate receptive from unreceptive females prevents males from carrying out costly courtship unnecessarily [1]. Individuals emit signals attractive to mates using different channels. Attractive cues can also be used as a phenotype of mate quality and can be visual [2], acoustic [3], tactile [4], or chemical [5]. Male chemical profiles communicate much information on its quality: symmetry, parasite/pathogen load, mate recognition and the quality of individual immune system ([6], [7] and references therein). Female chemical compounds can also be responsible for inducing male courtship behavior [8]. Sex pheromones that have been studied are indicative of mate location or receptivity but are also used for mate assessment [5].

Signaling their receptivity can also be beneficial for females in order to insure fertilization as well as to prevent male harassment when they are unreceptive. Higher attractiveness of receptive females have been observed in various species [9], [10], [11], [12]. Female receptivity is often signaled through chemical cues as in the snapping shrimp *Alpheus angulatus* where males are able to detect females approaching their receptivity period through distance chemical communication [13]. This can be expected as changes of physiological and reproductive status generally imply modifications of internal chemistry [1].

In crustaceans in particular, courtship behaviors can be very diverse from absent to very sophisticated. Mate choice can be the result of the female emitting signals attractive for males [14] or the opposite in other species [15] or mate encounter can occur just by chance [16]. Moreover, sexual pheromones and the impact of moulting cycles on females attractiveness have been reported in numerous crustacean species [17]. Mating and fertilization are closely associated with the female moulting cycle.

### Aggregation and Mate Choice

Spatial and temporal distribution of mates can impact mate choice and individual dispersion to find non-relative mates. Inbreeding avoidance is a major determinant of animal dispersal and mating patterns [18] although surprisingly in some species, it appears to be absent [19]. Gregarious species present a high risk of mating with relatives due to increased social interactions and attractiveness of conspecifics through aggregation pheromones [20], [21], [22], [23], [24]. However, mate choice for inbreeding avoidance has been described in the gregarious *Blattella germanica* [25].

In Oniscidea, aggregation is one of the numerous traits resulting from the adaptation of Crustaceans from aquatic to terrestrial life (brood pouch, conglobation posture…) [26], [27]. In terrestrial isopods, the aggregation pheromone is excreted via the faeces [28]. In the species *Ligia exotica, Porcellionides pruinosus* and *Armadillidium vulgare,* aggregation was shown to limit desiccation as well as to reduce metabolic rate and accelerate body growth [28]. The level of aggregation depends on the level of humidity in the environment and varies with the geographic location of populations [28], [29] but it is always at its highest during the period of reproduction. This co-occurrence suggests a role of aggregation in mate finding. Furthermore, aggregation directly affects reproduction because when females are in groups, and particularly with males, female vitellogenesis is accelerated [30], [31], [32] while female reproductive activity is synchronized through chemical cues transmitted via faeces [33]. In *Oniscus asellus*, female sizes affect their probability of being courted but mating success is independent of female and male size [34].

### Moulting and Reproductive Cycle

**Figure 1:**
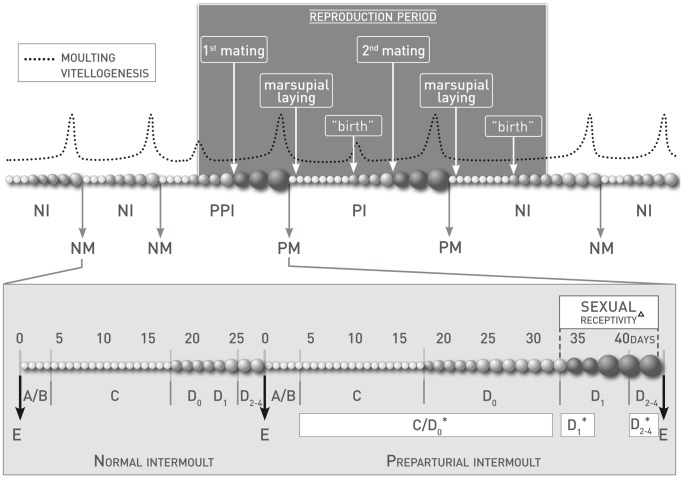
Moulting and reproductive cycle of terrestrial isopod females. Vitellogenesis. ––: approximate trend in the production of vitellogenin, o: oocytes (small white: before vitellogenesis, medium light grey: first stage of vitellogenesis, big dark grey: second stage of vitellogenesis); Moulting. NM: normal moult, PM: parturial moult, NI: normal intermoult, PPM: preparturial intermoult, PM: parturial moult; A/B: post-ecdysis, C: di-ecdysis, D_0_, D_1_, D_2–4_: beginning, middle and end of pre-ecdysis, E: ecdysis “birth” means the action of female releasing pulli from the brood pouch into the environment. ^Δ^: as described by Moreau and Rigaud [40]; *: experimental groups in our tests.

Female attractiveness is linked to their moulting status in many crustacean species [17]. In terrestrial isopods, female moulting and reproductive cycles are closely linked [35] and have been extensively studied. The secretory cycle of the Y-organ (also called moulting gland) corresponds to the moulting cycle and initiates pre-ecdysis [36]. Changes in the concentration of ecdysteroid secreted in the hemolymph mediate biochemical and physiological processes that occur during the moulting cycle [37]. Females can experience two types of moult: normal when non-breeding or parturial during the period of reproduction (respectively NM and PM in fig. 1). Indeed, the marsupium (brood pouch), necessary for egg incubation, only differentiates during these parturial moults. Moreover, complete ovarian maturation is only achieved during preparturial or parturial intermoult (PPM and PM in fig. 1) while during normal intermoult (NM in fig. 1) only the first stage of vitellogenesis occurs. The moulting cycle can be divided into five stages as described by Drach and Tchernigovtzeff [38] and adapted to terrestrial isopods by Steel [39]: i) A/B period or post-ecdysis represents about two days following the moult when animals neither move nor eat and are vulnerable to predation and desiccation; ii) C period or di-ecdysis is the period of maximal activity and it is also the period of incubation for breeding females; iii) D period or pre-ecdysis is the period during which ovarian maturation as well as hormonal and physiological changes occur. Indeed, calcium is stored in the form of white plates of calcium carbonate which begin to appear at the stage D_1_ ([39], [40]; see fig. 1). Details of calcium transport in terrestrial isopods has been previously described [41]. At the same time, the 2^nd^ stage of vitellogenesis begins for females preparing a parturial moult while the production of vitellogenin and ecdysteroids increases [42], [43]. During the stage D_2–4_, the calcium plates are larger and the production of vitellogenin and ecdysteroids is at the maximum [39], [42], [43]. The attractiveness of females varies a lot during their moulting and reproductive cycles [35]. Their maximum receptivity corresponds to the end of a preparturial or parturial intermoult when the second stage of vitellogenesis occurs [39], [40], [44]. If abiotic conditions are favorable, females can have two successive clutches during one breeding season either using only stored sperm or by remating after having released the first clutch [45].

The moulting cycle of *A. vulgare,* commonly named woodlice or pillbug (Figure S1), is very well described but its impact on chemical communication has not been investigated. *A. vulgare* excrete an aggregation pheromone and the results from some studies suggest that aggregation might also play a role in reproduction in this species [29], [30], [31], [32]. We used *A. vulgare* as a model species for studying chemical attractiveness between conspecifics depending on their sex and physiological status.

The aims of this study were to investigate sexual communication in *A. vulgare* and to test (i) if individuals perceive each others at short distance through chemoreception and (ii) if attractiveness of individuals varies depending on their sex and/or moulting status. We expected attractiveness to be higher between individual of the opposite sex but also to change through the moulting cycle.

## Materials and Methods

### Animal Rearing and Selection

Specimens of *Armadillidium vulgare* (Latreille, 1804) were derived from individuals collected in Nice, France in 1967. Specimens have been maintained in laboratory conditions ever since (20°C, natural photoperiod of Poitiers, France 46°40′N). Each spring since collection, gravid females have been isolated, offspring have been sexed and males and females separated in different boxes before sexual maturity. The breeding has been controlled in order to avoid inbreeding. Individuals are maintained in plastic boxes (26×13 cm) on moistened compost and they are fed with slices of fresh carrots and dried leaves of linden (*Tilia* sp.) *ad libitum*.

As experience can influence female preference both in vertebrates and invertebrates ([46] and references therein), we used one-year old virgin individuals of similar size and sexually mature (size >0.7 mm; see [47]). Females were also sorted according to their physiological status (see fig. 1). They were tested either when in di-ecdysis or at the beginning of pre-ecdysis, before the appearance of the white plates (C/D_0_ period), at the appearance of the white plates (middle of pre-ecdysis or D_1_ period) or with advanced white plates (end of pre-ecdysis or D_2–4_). Males were all tested in di-ecdysis or beginning of pre-ecdysis (C/D_0_). All tested females were in preparturial intermoult. Their physiological status were confirmed post-experimentally by the shape of the calcium plates [40].

### Choice Chamber

**Figure 2:**
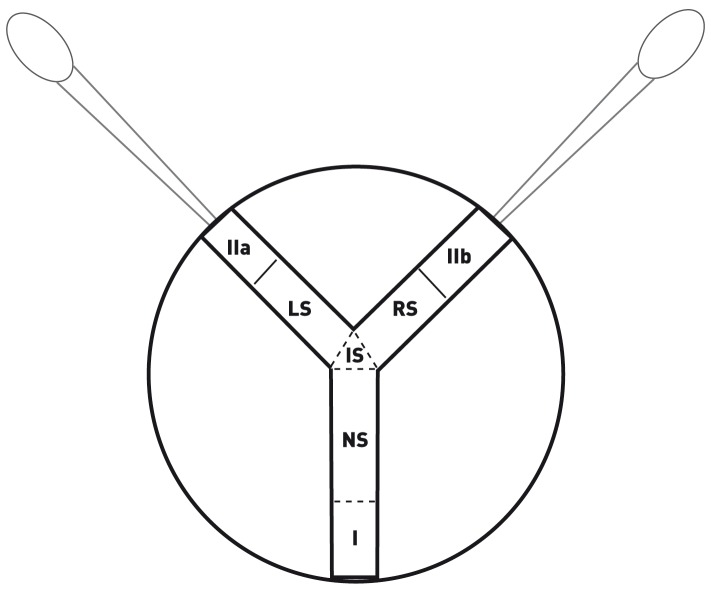
Schematic view of the Y-shaped choice chamber used to test the detection of conspecifics by short-distance chemoreception in *Armadillidium vulgare* (I: initial position of tested individual, IIa and IIb : position of target individuals; NS: Neutral Section, IS: Intermediate Section, LS: Left Section, RS: Right section) with two plastic pipettes placed at the end of each tunnels and used to pulse air regularly into the system.

To test the attractiveness of conspecifics in *A. vulgare*, we used a Y-shaped choice chamber (fig. 2) built in a plastic Petri dish (9.5 cm diameter) covered with a filter paper renewed between each experiment to avoid the presence of retained odours. The plastic elements of the device were cleaned using alcohol everyday. Rigid plastic tunnels were used, with two of them separated in two sections by a mesh in order to create the zones (IIa) and (IIb). This mesh was covered by an opaque paper with tiny holes so that the air could pass through it whilst preventing visual contact between the individuals. These papers were also changed between each experiment. Moreover, two plastic pipettes were sealed at the end of these tunnels to ensure gas exchange with the outside and were used to pulse air regularly (two light pressures at the beginning of the experiment and two others five minutes later) into the system passing through the sections (IIa) and (IIb) to spread the odour of the animals presented for choice.

At the beginning of the experiment, the isopods were in the sections located at the three extreme parts of the tunnels: either one or two target individuals were placed in sections (IIa) and (IIb) (one in each section) and the tested specimen was placed in section (I) and was then able to move into four other sections. The position of target individuals in (IIa) and (IIb) sections were inverted between each experiment so that a preference for the right or left side was not likely to bias the results. Moreover, the target and tested individuals were used only for one test. The target animals were placed in their section 15 minutes before the experiment began so that they got accustomed to the device. The tested individual was then carefully transferred from the group-sorted box to position (I) inside the choice chamber. From the moment the tested specimen entered the neutral section (NS), the time spent in each section and particularly in the left (LS) and the right sections (RS) joining respectively to (IIa) and (IIb) sections was monitored. This was performed by recording the tested individual behaviour using the program EthoLog 2.2 [48]. Each test lasted for 10 minutes.

### Experimental Groups

Experiments were realized in spring 2009 and 2010, during the natural mating period, always between 2pm and 5pm, in the same conditions of light, temperature and humidity to which animals were raised in (1 Lux, 20°C, 60% ±10%).

In the first experiment, the tested individual was placed in a device where a section housed a conspecific while the other was empty (alternatively in section IIa and IIb). In total four combinations were used (Table 1, exp. 1) to test male and female preference between an empty section and a target individual, male or female. Each test was repeated 20 times with different individuals. All the females were in di-ecdysis or at the beginning of pre-ecdysis (C/D_0_ period) (Figure 1).

**Table 1:** Summary of the different combinations tested and number of replicates (N) for each.

		IIa or IIb					
		Exp. Choice 1		Exp. Choice 2			
Target animalTested animal		Male	Female C/D_0_	Male/Female C/D_0_	Male/Female D_2–4_	FemaleC/D_0_/Female D_2–4_	FemaleC/D_0_/Female D_1_
I	Male	N = 20	N = 20	N = 20	N = 20	N = 25	N = 10
	Female C/D_0_	N = 20	N = 20	N = 20			
	Female D_2–4_				N = 20		

(C/D_0_: di-ecdysis and beginning of pre-ecdysis (no calcium plates), D_1_: middle of pre-ecdysis (appearance of calcium plates), D_2–4_: end of pre-ecdysis (advanced calcium plates), see fig. 1).

In the second experiment, the tested individual was introduced in a device where 2 target individuals were placed in the isolated sections (IIa and IIb) for each test. We tested the preferences of males and females for conspecifics depending on their sex and physiological status (Table 1, exp.2). We tested male attractiveness between a section with a target male and a target female (either in advanced di-ecdysis, stage C/**D_0_** or at the end of pre-ecdysis, stage D_2–4_). Male choice between two females at different moulting stages was also tested with either (i) an early di-ecdysis female **C**/D_0_ and a pre-ecdysis female D_2–4_ with advanced white calcium plates (Figure 1) or (ii) a di-ecdysis female C/D_0_ and a pre-ecdysis female D_1_ with appearing white calcium plates (alternatively in section IIa and IIb). Choice test with early and advanced di-ecdysis female C/D_0_ were defined at the end of the experiment, when females moult. We also tested the choice of females between a target female in the same moulting stage and a target male. This test was performed for both C/D_0_ females and D_2–4_ females (Figure 1).

In the third experiment, the tested individual had to choose between a chemical extract of one individual (C/D_0_) placed on inorganic item (a piece of odour-free eraser) fully covered with aluminum and another item only covered by solvent. Chemical extraction was obtained from a total of 20 females. Twenty females were first killed through freezing. Two sets of 10 females were then each submerged in 5 ml of dichloromethane for 24 hours. The remaining solution was then used as chemical extract. The behavioral experiment was repeated 10 times.

### Statistical Analysis

The time spent by the tested individual in the right and left sections, was compared using the non-parametric Wilcoxon test for dependent samples. The attractiveness of target individuals was calculated as the median of the time (in seconds) spent by the tested individuals in front of their section (i.e. in the right or left section).

## Results

We conducted behavioural choice tests in order to compare the attractiveness of conspecifics depending on their sex and physiological status in the terrestrial isopod *A. vulgare*. Here we present the time that the tested individuals spent in the right and left sections which face the sections housing target conspecifics.

### Choice 1: Empty Section Versus Conspecific

**Figure 3:**
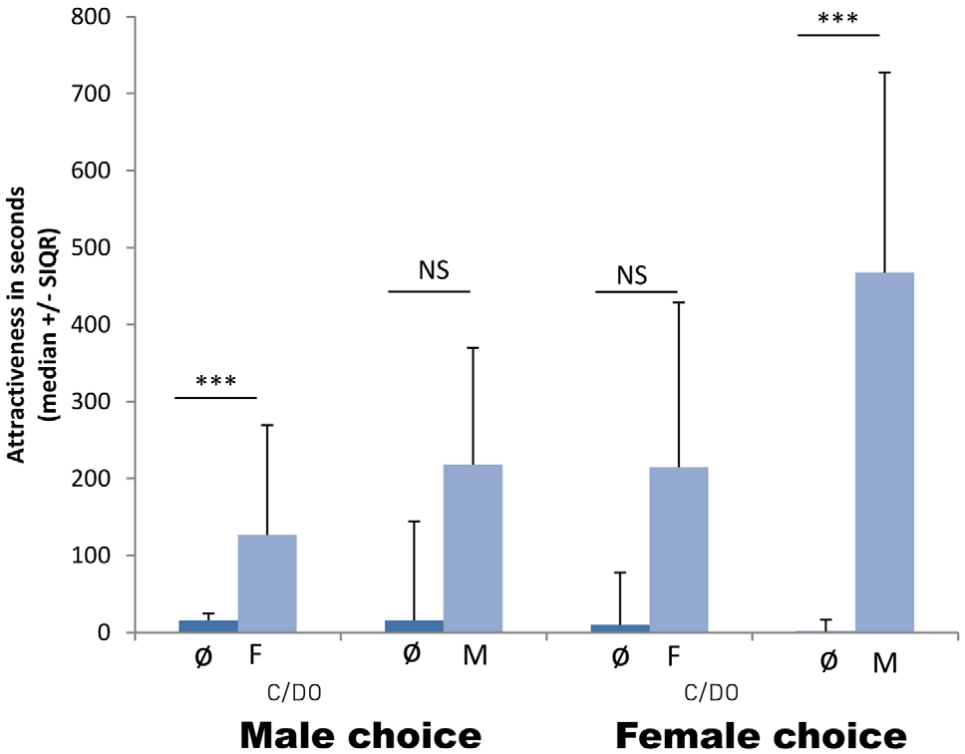
Time spent by males and females in the right (RS) and left (LS) sections depending on the presence or absence of conspecific in the adjacent section. (F: female, M: male; C/D_0_ : di-ecdysis and beginning of pre-ecdysis (no calcium plates), Ø = empty section with no individual, see fig. 1; NS: non-significant p≥0.05, ***: p<0.001).

Both males and females spent significantly more time in front of the section harbouring a conspecific of the opposite sex (respectively: Wilcoxon: *T* = 26, *N* = 20, *P* = 0.0016 and *T* = 14, *N* = 20, *P*<0.0001; figure 3). In contrast, when presented with a conspecific of the same sex, they did not show any preference for the inhabited section (respectively: Wilcoxon: *T* = 72, *N* = 20, *P* = 0.109 and *T* = 66, *N* = 20, *P* = 0.07; figure 3).

### Choice 2: Discrimination between Conspecifics

#### Male choice

**Figure 4:**
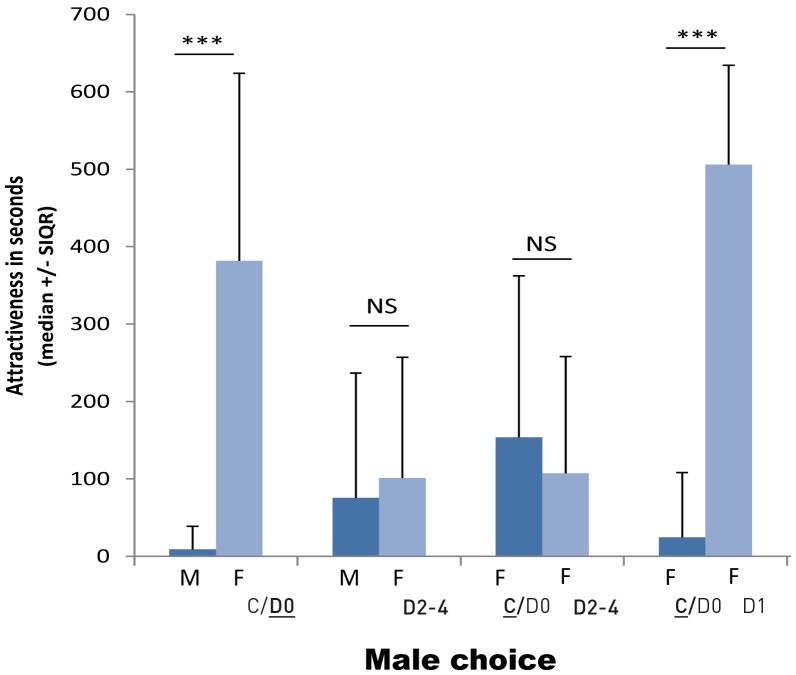
Time spent by males in the right and left sections depending of the sex and the moulting status of the conspecific present in the adjacent section. (F: female, M: male; C/D_0_ : di-ecdysis and beginning of pre-ecdysis (no calcium plates), D_1_: middle of pre-ecdysis (appearance of calcium plates), D_2–4_: end of pre-ecdysis (advanced calcium plates), see fig. 1; NS: non-significant p≥0.05, ***: p<0.001).

Males spent significantly more time in front of the section housing a female in advanced di-ecdysis (C/**D_0_**) than in front of the section with another male (Wilcoxon: *T* = 36, *N* = 20, *P* = 0.005; figure 4). However, this was not observed when the target female was in pre-ecdysis *i.e.* with advanced white plates and calcium deposit (D_2–4_) (Wilcoxon: *T* = 98, *N* = 20, *P* = 0.397; figure 4). When the male had to choose between a female without white plates in early di-ecdysis (**C**/D_0_) and one in pre-ecdysis (D_2–4_), there was no significant preference (Wilcoxon: *T* = 131, *N* = 25, *P* = 0.198; figure 4). However, when the choice test was between a female in di-ecdysis (C/D_0_) and a female with appearing white plates (D_1_) the males spent significantly more time in the section close to the female D_1_ (Wilcoxon: *T* = 3, *N* = 10, *P* = 0.0063; figure 4).

#### Female choice

**Figure 5:**
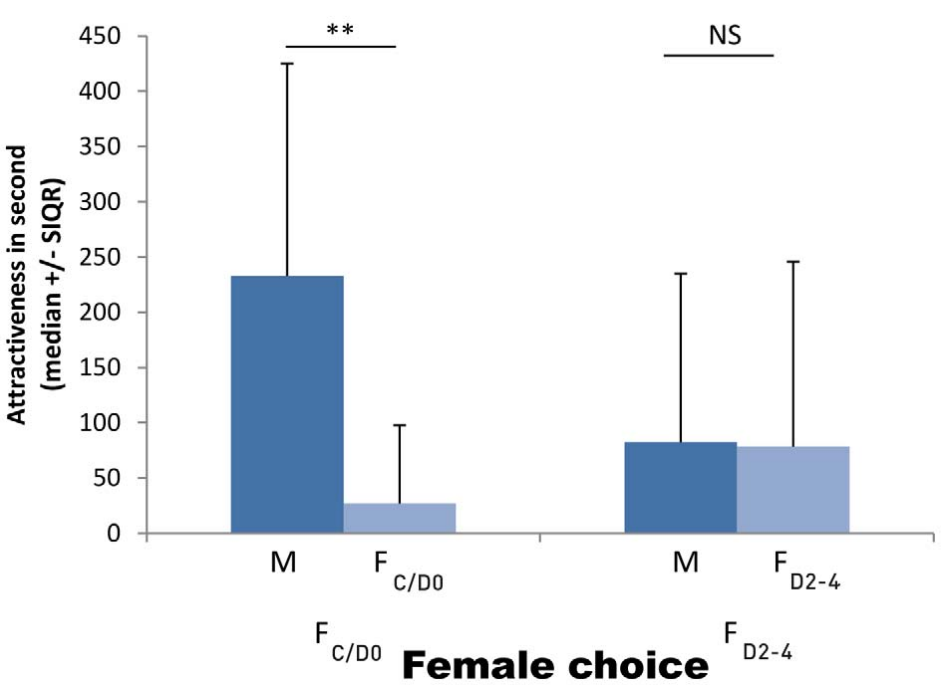
Time spent by females at different moulting stages in the right (RS) and left (LS) sections depending of the sex of the conspecific present in the adjacent section. (F: female, M: male; C/D_0_: di-ecdysis and beginning of pre-ecdysis (no calcium plates), D_2–4_: end of pre-ecdysis (advanced calcium plates), see fig. 1; NS: non-significant p≥0.05, **: p<0.01).

Females in di-ecdysis (C/D_0_) had a significant preference for the section housing a male rather than for the section housing another female also in di-ecdysis (C/D_0_) (Wilcoxon: *T* = 57, *N* = 20, *P* = 0.036; figure 5). However, there was no significant preference when the tested female was in pre-ecdysis (D_2–4_) or when both male and female were in pre-ecdysis (D_2–4_) (Wilcoxon: *T* = 97, *N* = 10, *P* = 0.382; figure 5).

### Choice 3: Male Choice between Female Chemical Extract and Pure Solvent

**Figure 6:**
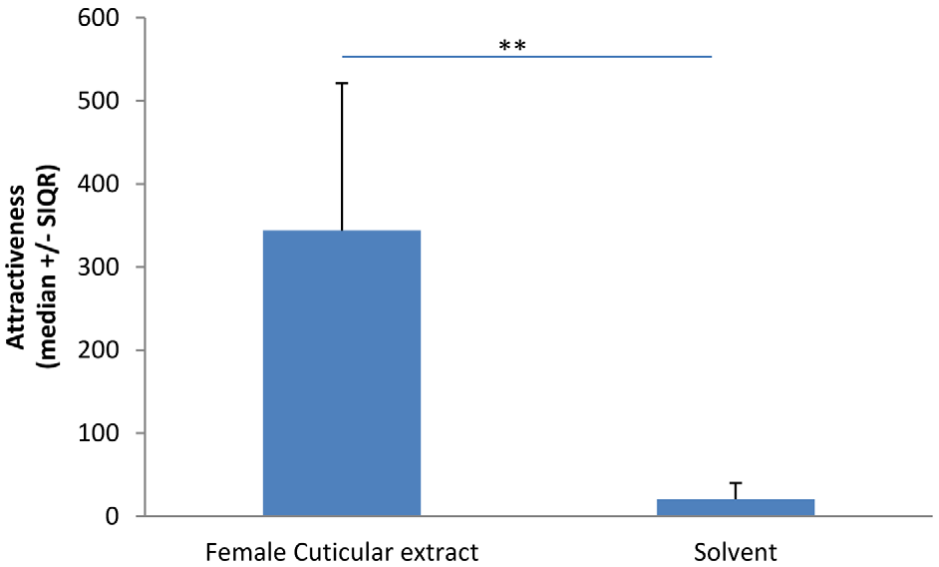
Time spent by males in the right and left sections depending of presence or not of female cuticular chemical extract. (**: p<0.01).

Males spent significantly more time in front of the section containing an item covered with female extract than in front of the section containing an item covered with solvent only (Wilcoxon: *T* = 64, *N* = 10, *P* = 0.002; figure 6).

## Discussion

### Aggregation Pheromone and Individual Affinity

Our work revealed an individuals' ability to perceive conspecifics based on olfaction in *A. vulgare*. Both males and females were significantly more attracted to a conspecific of the opposite sex when the other section was empty. In contrast when they had to choose between a conspecific of the same sex and an empty section there was no significant difference in the time they spent near either sections. Thus we highlight that individual attraction is mainly sex dependent and that the aggregation pheromone is not attractive to all individuals in our experimental design.

Aggregation enhances the emergence of basic cooperation [49] and social interactions between conspecifics [50]. Aggregation resulting from inter-attractiveness is usually not restricted to individuals of the opposite sex and on the contrary, individuals of the same sex are attracted to each other. In terrestrial isopods, aggregation as a response to pheromones released by conspecifics has been shown in several species *A. vulgare, Oniscus asellus, Porcellio scaber* [51]. Using a Y-maze olfactometer, Takeda [28] demonstrated that chemical compounds secreted in the gut and released in faeces constitute an aggregation pheromone for *A. vulgare.* Since then, most studies on aggregation did not focus on the cues inducing aggregation but on the biotic and abiotic factors influencing its intensity [30], [52], some authors even speaking of “passive aggregation” due to individual environmental preference for most favorable micro-habitats [27]. But two recent studies demonstrate that inter-attractiveness between *P. scaber* conspecifics is stronger than individual preferences which only impacts on the location of aggregates [53], [54]; however the authors did not determine the nature of the attractive cues. In another terrestrial isopod species *O. asellus*, perception of conspecifics seems to be possible by substratum-bound info-chemicals while sexes are mainly distinguished upon antennal contact [34].

Next, we demonstrated that male preference and attraction were significantly higher for the opposite sex. Moreover, males discriminated between females at different moult stages and preferred females at the beginning of pre-ecdysis with appearing white plates (D_1_) compared to other females at earlier or later stages of their moult cycle. Therefore we suggest that perception cues are air-borne info-chemicals that enable the perception of conspecifics, their sex and even their moulting status in the terrestrial isopod *A. vulgare*. If vision can be well developed in some Crustaceans and used in communication [55], [56], visual acuity seems likely to be extremely reduced in terrestrial forms but could still be used for mate choice. Studies on orientation and relocation of food patches with *A. vulgare* suggest that both vision and olfaction are used in these processes but this has not been demonstrated yet [57]. In any case, our experimental setup would not allow visual perception of the conspecifics. As for acoustic communication, vibration quality or stridulation (duration and intensity) can be used to discriminate mate quality [58], [59]. In terrestrial isopods, stridulations appear to be used for defense, as it occurs only when individuals are partially or totally rolled-up and had been only described in *Armadillo officinalis* [60]. We find it more likely that the attractiveness of the different individuals tested was determined by their odour. In another terrestrial isopod species, *O. asellus*, chemical cues were shown to signal the presence of conspecifics and to increase male activity in areas previously marked by females [34]. To demonstrate the role of olfaction, we extracted the female odours and applied them on inorganic items. Female odour extract attracted males without other stimulated cues. The next step would be to check whether non-attractive individuals become attractive after coating them with the odour of attractive individuals.

Physiological changes are commonly related with individual odour changes. The attractiveness increase across intermoult phase could be linked with physiological development and odour variation. Numerous changes occur during the second stage of vitellogenesis such as an increasing production of vitellogenin and other proteins [43], the release of ecdysteroids [42], and the translocation of calcium [61], which could induce a change of odours and attractiveness. The production of 20 hydroxyecdysone (20 HD) is linked to ovary development and it functions as a pheromone for some species, but recent evidence shows that this is not as straightforward [17]. For example 20 HD is strongly associated with individual’s moult cycle and plays a role in the pheromone involved in reproductive behaviour of the shore crab *Carcinus maenas*, but does not initiate pair formation [62].

### Aggregation as the First Step to Mate Finding?

The first function of aggregation in Oniscidea is most likely to limit desiccation [27]. However in species well adapted to terrestrial life, as in *A. vulgare*, the thick cuticle and conglobation behaviour are already very effective to prevent water loss [27]. Aggregating behaviour may be conserved for additional benefits which could actually be linked to reproduction. Indeed, aggregation can permit an acceleration of vitellogenesis and the synchronization of moult cycles in females when they are in a group and even more when males are present [30], [31], [32]. Aggregation could reduce the time spent on finding mates and increase mating opportunities. Indeed, we found that even if all conspecifics were attracted to each other, individuals from the opposite sex were the most attractive. As a consequence, inside aggregates, chemical cues could be used to find potential mates.

**Figure 7:**
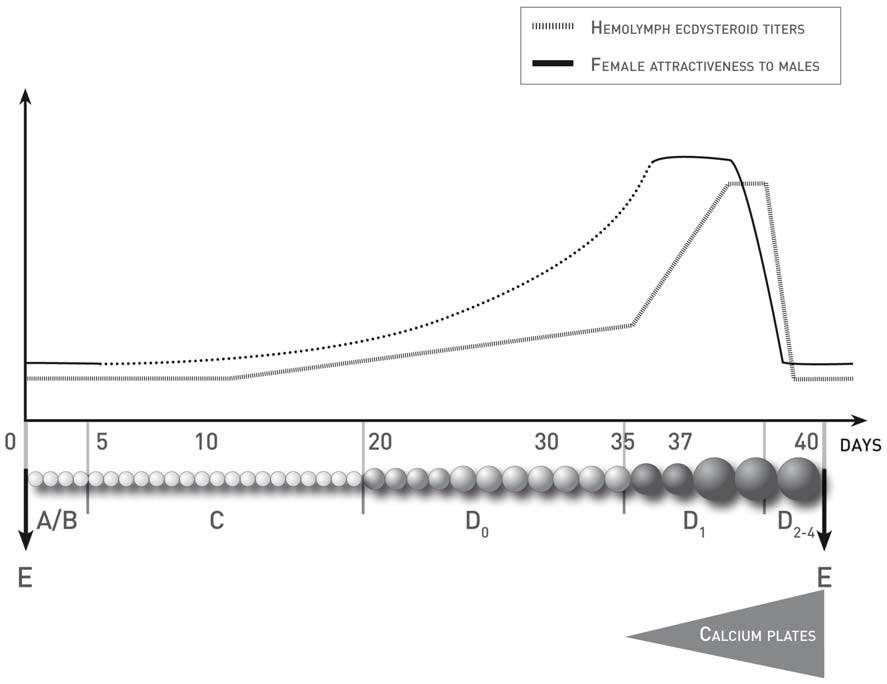
Variation of female attractiveness to males throughout a female intermoult preceding a parturial moult. (o: oocytes (small white: before vitellogenesis, medium light grey: first stage of vitellogenesis, big dark grey: second stage of vitellogenesis); A/B: post-ecdysis, C: di-ecdysis, D_0_, D_1_, D_2–4_: beginning, middle and end of pre-ecdysis, E: ecdysis; triangle: appearance and development of white calcium plates; the evolution of attractiveness during di-ecdysis is approximated according to the difference that was observed between attractiveness of post-ecdysis and pre-ecdysis females). The hemolymph ecdysteroid titers curve is adapted from Chang and Mykles [37]. The female attractiveness to males curve is based on the current results (plain lines) and unknown attractiveness evolution (dots).

On the other hand, aggregation might also increase male-male competition. Indeed, in *A. vulgare*, female receptivity is limited to a very short period while males are sexually active all year round except when moulting [44]. In aquatic species, pre-copulatory mate-guarding is a strategy by which males can monopolize a female mate [63], [64]. However, in terrestrial isopods such as *A. vulgare*, this strategy is not observed. Several explanations have been proposed for this difference including higher costs of mate-guarding in terrestrial compared to aquatic environment, male-induced female resistance and capacity of sperm storage by females in terrestrial species [63], [65]. Indeed, sperm can be stored for 12 months by females *A. vulgare* and be used to fertilize another clutch [45], [66], [67]. Even if the female remates with a second male, at least 50% of the brood will be sired by the first male due to stored sperm precedence. In *A. vulgare*, mating induces an immediate refractory period during which females reject male mating attempts [66]. All these parameters make it very beneficial for a male to locate receptive females before any other male. This is congruent with the hypothesis of Lefebvre *et al.* [68] about antennal sexual dimorphism being a consequence of male scramble competition. Here we show that males can locate receptive females, using short-distance chemical cues. Indeed, we found that females starting the second stage of vitellogenesis (start of pre-ecdysis or D_1_) were the most attractive, which matches their period of higher receptivity (Figure 7). On the other hand, females at the end of pre-ecdysis (D_2–4_) were less attractive than other females and they were not particularly more attracted to males than to other females. Eggs are fertilized when laid in the marsupium after the parturial moult, by passing through the oviduct where the sperm is stored [67]. Males could be less attracted to females at this stage because they are more likely to have already mated and stored sperm. These females might as well not be receptive anymore and resist mating attempts because of the refractory period. Moreover, moulting individuals are likely to be more vulnerable (predation, infection) and might not be so attracted by conspecifics compared to the rest of the time when they are gregarious.

Finally we showed that female preference and attraction were higher for the opposite sex but this preference depended on her physiological status. Females in pre-ecdysis (D_2–4_) did not prefer males to other females at the same moulting stage. Individual behavior changed during the moult cycle and both males and females were attracted by the opposite sex. In *A. vulgare* sexual selection could be made by both sex and a possible adaptive consequence of female preference has not been studied yet.

We conclude that *A. vulgare* can perceive and discriminate between male and female conspecifics as well as their physiological status based on short-distance chemical perception only. Aggregation which is at its highest level during the period of reproduction would promote physiological synchronization and even accelerate ovary maturation [30] to reach sexual receptivity. Thus, during the mating season, it would favour mate finding/prospection and choice. The increasing female attractiveness across the moulting cycle might encourage males to remain close to females until they were sexually receptive. Additional behavioural tests coupled with chemical analysis of cuticle compounds will enable the chemical compounds that elicit individual attractiveness and female sexual receptivity to be characterized.

## Supporting Information

Figure S1 Picture of terrestrial isopod*s A. vulgare,* commonly named woodlice or pillbug. ©F.-J. Richard.
(TIF)
