# Supplementary figures and images for: The Best Timing of Mate Search in Armadillidium vulgare (Isopoda, Oniscidea)
Source: PLoS One. 2013 Mar 1;8(3):e57737. doi: 10.1371/journal.pone.0057737 (PMC3585876; doi:10.1371/journal.pone.0057737)

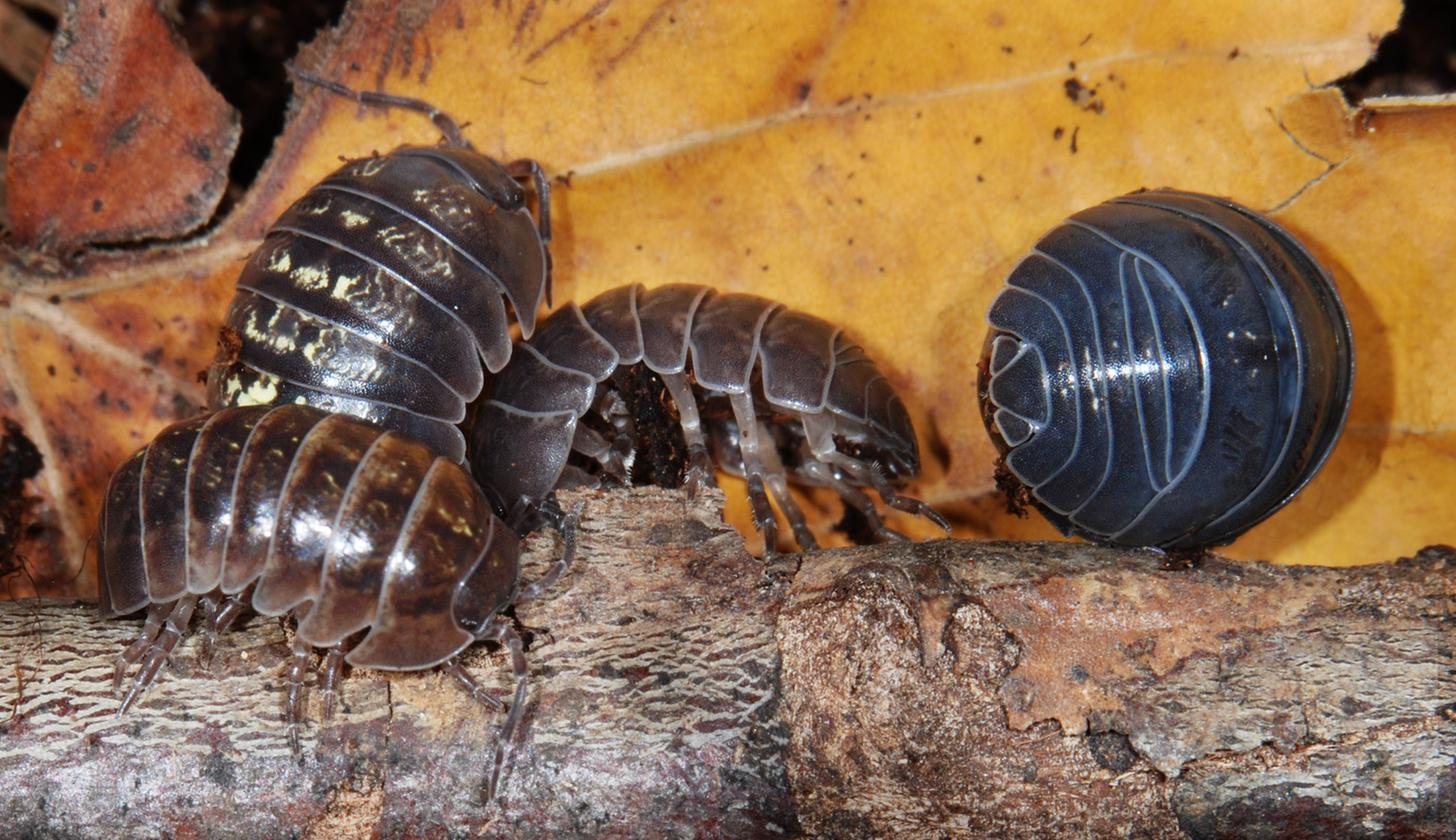

Supplement: Figure S1 — Picture of terrestrial isopods A. vulgare, commonly named woodlice or pillbug. ©F.-J. Richard. (TIF) [file pone.0057737.s001.tif]
